# A photodynamically sensitized dendritic cell vaccine that promotes the anti-tumor effects of anti-PD-L1 monoclonal antibody in a murine model of head and neck squamous cell carcinoma

**DOI:** 10.1186/s12967-022-03707-x

**Published:** 2022-11-03

**Authors:** Shuang Li, Ding Wang, Jinzhang Cheng, Jicheng Sun, Dhan V. Kalvakolanu, Xue Zhao, Di Wang, Yunhan You, Ling Zhang, Dan Yu

**Affiliations:** 1 Department of Otolaryngology-Head and Neck Surgery, The Second Hospital of Jilin University, No. 218, Ziqiang Street, Nanguan District, 130041 Changchun, Jilin Province People’s Republic of China; 2 Key Laboratory of Pathobiology, Department of pathophysiology, College of Basic Medical Sciences, Ministry of Education, Jilin University, 126 Xinmin Street, 130012 Changchun, Jilin P.R. China; 3 Greenebaum NCI Comprehensive Cancer Center, Department of Microbiology and Immunology, University of Maryland School Medicine, Baltimore, MD USA

**Keywords:** Head and neck squamous cell carcinoma, PD-L1, Immunotherapy, Immune checkpoint, DC vaccine, Photodynamic therapy

## Abstract

**Background:**

Immune checkpoint inhibitors are promising tools in combating several cancers, including head and neck squamous cell carcinoma (HNSCC). However, a substantial portion of HNSCC patients do not respond to PD-L1 antibody. Here we describe a photodynamic therapeutic (PDT) approach to enhance anti-tumor effects of the anti-PD-L1 antibody.

**Methods:**

Phototoxicity of PDT was confirmed using fluorescence microscopy, Cell Counting Kit-8 (CCK-8), Enzyme Linked Immunosorbent Assay (ELISA) and flow cytometry analyses. Phenotypic and functional maturation of immature DCs (imDCs) induced by PDT were measured using flow cytometry and ELISA. A mouse model was established using the HNSCC line, SCC7, and was used to evaluate therapeutic effects of PDT-DC vaccine in facilitating anti-tumor immunity of PD-L1 antibody.

**Results:**

Immunogenic cell death (ICD) of SCC7 cells was induced by PDT with 0.5 *µ*M of m-THPC and the 5 J/cm^2^ of light dose. ICD of SCC7 cells stimulated imDCs maturation. In vivo assays suggested that PDT-DC vaccine and anti-PD-L1 mAb synergistically induced anti-tumor immunity and suppressed tumor progression.

**Conclusion:**

PDT-DC vaccine enhances therapeutic effects of PD-L1 antibody, which might provide a novel approach for HNSCC immunotherapy.

**Graphic abstract:**

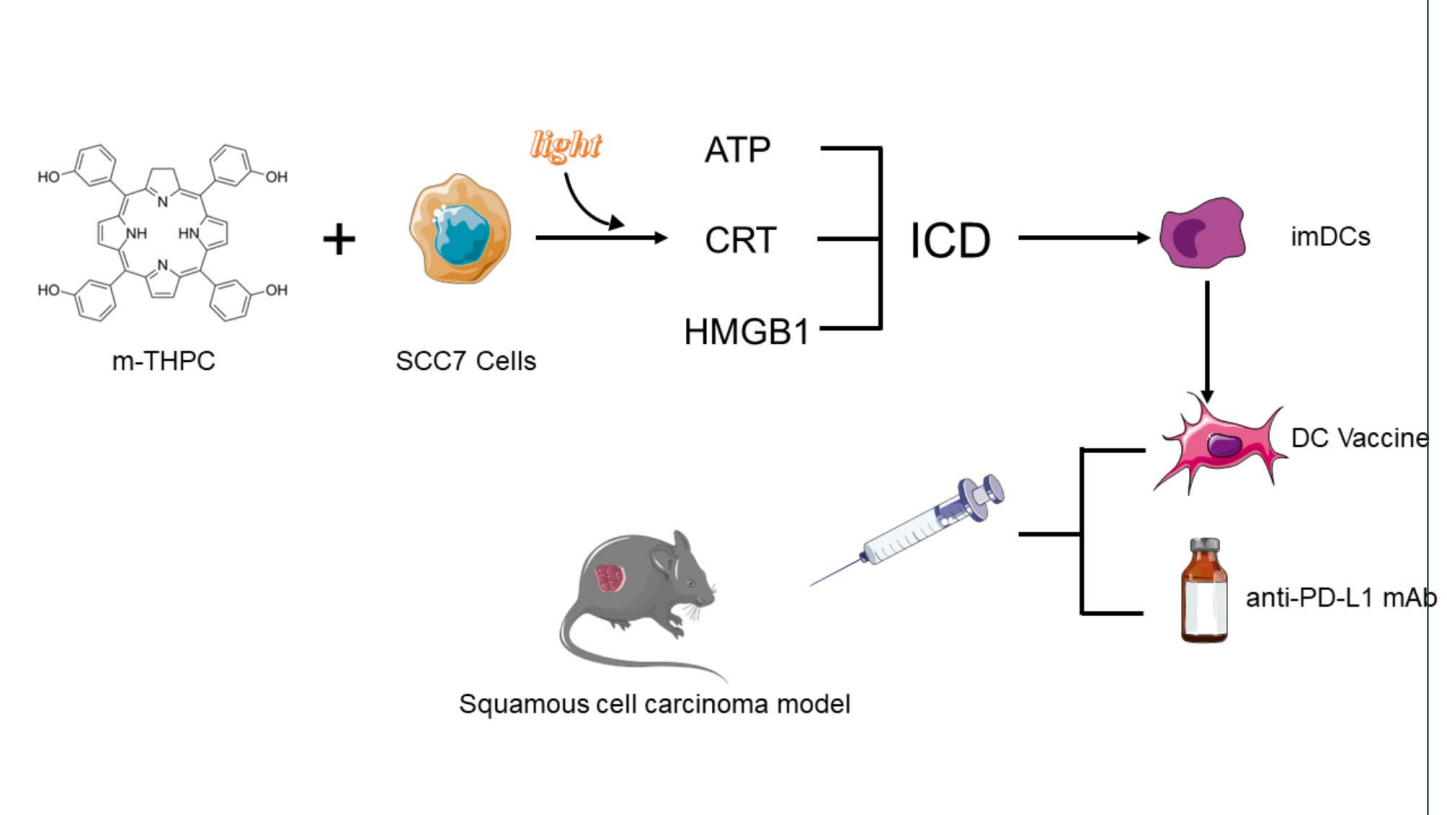

## Introduction

Head and neck cancer squamous cell carcinoma (HNSCC) rank as the sixth most common cancer worldwide. Approximately 835,000 new cases and 431,000 deaths due to HNSCC were reported globally in 2018[1]. Its etiology can trace to major factors like the continuous tobacco exposure, alcohol consumption, betel chewing, and virus infection. Although intensive multimodality managements are performed on HNSCC, little improvement of its five-year survival rate has been noted in the recent decades[2]. Due to the unique anatomical structures in the head and neck area, recurrence or metastasis often occur in HNSCC which is the main reason for patient death[3]. Thus, there is a greater need to find new therapies for HNSCC treatment.

Recently, immunotherapy has received an unprecedented attention due to the success of immune checkpoint inhibitors, such as anti-CTLA4[4], anti-PD-1[5] and anti-PD-L1[6] which have been confirmed in multiple clinical trials, including recurrent and metastatic HNSCC[7]. However, these immune checkpoint inhibitors are only beneficial to a subset of patients at least partially because of dysfunctions of CD8^+^ cytotoxic T-lymphocytes (CTL)[8]. Moreover, several studies indicated that HNSCC cells are unable to be recognized by CTL in vitro[9].

Dendritic cells (DCs) as the most professional antigen-presenting cells (APCs) have a unique role in activating CTLs against tumor. To this end, various DC vaccines have been used in animal models[10, 11]. Currently, a number of stimulants (such as chemokines[12], tumor antigen fusion protein[13], and tumor antigen peptide[14]) have been tested for promoting the efficacy of DCs, which have made great advancements in DC vaccines. However, a majority of them have not met with the required safety and efficacy levels for clinical use[15]. Thus, a search for new methods to facilitate antigen-presenting function of DCs remains of great significance.

Photodynamic therapy (PDT) is an emerging minimally invasive approach, which has been approved by US Food and Drug Administration for the treatment of some cancers and benign diseases[16]. It is based on interaction between photosensitizers and light of a specific wavelength. This procedure contributes to reactive oxygen species (ROS) generation and finally cell death. The early photosensitizers, such as eosin red and the hematoporphyrin derivatives, were not widely used for clinical PDT due to their side effects. 5,10,15,20-Tetrakis (3-hydroxyphenyl) chlorin (m-THPC or, Temoporfin) is one of the most effective the second generation of photosensitizers[17, 18], which is applied at a lower concentration and light dose than other clinically approved photosensitizers for PDT response[18–20]. Notably, m-THPC was approved for the HNSCC treatment in Europe[19, 21]. Recently, it has been suggested that PDT induces a specific type of immunogenic cell death (ICD) in several cancer cells[22–24]. ICD manifests as a release of damage-associated molecular patterns (DAMPs), and mitochondrial dysfunction and endoplasmic reticulum stress induced by ROS[25] which leads to further antigenic spread[26] and DC recruitment[27]. In high grade glioma, Grag et al. have vaccinated orthotopic glioma mice with PDT induced ICD-based DC vaccines, which show an anti-glioma protective immunity by pulsing DC maturation and functional activation[28]. Thus, PDT has the potential to strongly activate antigen-presenting DCs.

Mutated tumor specific antigens, generated from the inherent genetic instability and high mutagenic rate of tumors, play a prominent role in tumor immune escape by failing the T cell response [29]. HNSCC can evade immune surveillance via downregulating immunogenicity and activating immunosuppressive pathways due to its high-level expression of tumor specific neoantigen expressions [30, 31]. Hence, efficient recognitions of HNSCC-specific antigens mediated by T cell are crucial for HNSCC immunotherapies and new approaches are still needed for improving immune responses to HNSCC. These findings inspired us to develop a strategy involving ICD induced by PDT, which was able to induce maturation and activation of DCs efficiently. We also aimed to demonstrate PDT-DC vaccine in promoting PD-L1 monoclonal antibody (mAb) therapeutic efficacy in a mice model of squamous cell carcinoma.

## Materials and methods

### Cell lines and cell culture

The SCC7 cells were obtained from FuHeng Biology (Shanghai, China). The authenticity of cell lines was ensured by the supplier using STTR markers.SCC7 cells were maintained in RPMI-1640 medium (Gibco, USA) supplemented with 10% fetal bovine serum (FBS; FuHeng Biology, Shanghai, China), 100 units/mL of penicillin (HyClone, South Logan, UT), and 100 µg/mL streptomycin (HyClone) at 37 ℃ and 5% CO_2_.

### Cellular uptake of m-THPC

SCC7 cells were cultured in black 96-well microplates with a clear glass bottom (Corning, USA) at a density of 6,000 cells/well. After reaching a confluence of 60–70%, the cells were washed twice with PBS and then incubated with m-THPC (MedChem Express, NJ, USA) of different concentrations (ranging from 0.5 to 10 *µ*M) in serum-free medium in the dark for 24 h. SCC7 cells without treatment were used as a negative control. Intake of m-THPC by the SCC7 cells was detected using an inverted fluorescence emission microscope (IX71, Olympus, Japan) and quantified using a microplate reader (FLUOstar Omega, BMG LABTECH GmbH, Ortenberg, Germany). The fluorescence excitation was set at 460 nm and the emission was monitored at 650 nm. The fluorescence intensity was corrected for autofluorescence background (n = 5). CCK-8 assay kit (Sangon Biotech, Shanghai, China) was performed to detect cell viability of SCC7 cells according to the manufacturer’s instructions and the optical density was measured at 450 nm with microplate reader.

### PDT-induced ICD

SCC7 cells were incubated in the dark with m-THPC at indicated concentration for 24 h, then irradiated using a laser beam (Changchun Institute of Optics, Fine Mechanics & Physics, Chinese Academy of Sciences, Changchun, China) at 635 nm with different light doses (0.1 to 5 J/cm^2^) in photosensitizer-free media, SCC7 cells without irradiation were used as a negative control. Cell death was analyzed by CCK-8 assay after 24 h.

### ICD assays

For detecting ICD, SCC7 cells (3 × 10^5^/plate) were seeded in 35 mm dishes (NEST, Jiangsu, China), and PDT was performed as described above. After 1 h of PDT, SCC7 cells suspensions were treated with anti-calreticulin (CRT) antibody (ab196159, 0.5 mg/ml), then incubated for 1 h at room temperature. The assay was run on a BD FACSCalibur flow cytometer. Supernatants were harvested for ATP quantification (Enhanced ATP assay kit, Beyotime Biotechnology, Shanghai, China) and high mobility group protein B1 (HMGB1) measurement by ELISA (Meimian industrial Co., Ltd, Jiangsu, China) after 4 and 24 h of PDT, respectively. All assays were performed as described by respective manufacturers and measured on a microplate reader (FLUOstar Omega, BMG LABTECH GmbH, Ortenberg, Germany).

### Generation of DCs in vitro

Bone marrow was obtained from the femur and tibia of multiple syngeneic C3H/HeN mice. After depleting erythrocytes, cells were seeded at 1.5 × 10^6^ cells/well in 24-well plates with RPMI-1640 supplemented with 10% FBS, 20 ng/ml granulocyte macrophage colony-stimulating factor (GM-CSF, PeproTech), 20 ng/ml interleukin-4 (IL-4, PeproTech) 100 U/mL of penicillin, and 100 µg/mL streptomycin. On day 2 and 4, 50% of the media were replaced with fresh media containing GM-CSF and IL-4. On day 6, immature DCs (imDCs) were isolated for experiments.

### Analysis of DCs in vitro

SCC7 cells were killed using either mTHPC -PDT or three cycles of freeze-thaw lysis. Killed SCC7 cells were then cocultured with imDCs at a 1:1 ratio for 24 h. As a positive control, imDCs were stimulated by 500 ng/ml of E. coli lipopolysaccharide (LPS, Sigma); Maturation of DCs was analyzed using immunofluorescent anti-mouse CD11c PE-Cy7 (117,318, N418, Biolegend) and anti-mouse MHC-II APC (107,614, M5/114.15.2, Biolegend) in accordance with the manufacturer’s instructions. Samples were then collected and analyzed using a BD FACSCalibur flow cytometer. After co-incubating imDCs with the m-THPC-PDT-SCC7 cells, the supernatants were harvested for interleukin-12 (IL-12) and interleukin-6 (IL-6) measurement using ELISA (Meimian industrial Co., Ltd, Jiangsu, China).

### Animal experiments

C3H/HeN mice (female, 6-8weeks old purchased from Vital River Laboratory Animal Technology Co Beijing, China) were challenged subcutaneously on the right flank with 5 × 10^6^ live SCC7 cells. On day 1 (24 h after inoculation), tumor-bearing mice were randomly divided into four groups (n = 5). On day 3 after tumor implantation, mice were assigned into four different treatment groups: (A) control group: 0.1 ml PBS were injected subcutaneously around peritumoral regions plus 100 µg/mouse of IgG2b isotype (MPC-II, BioXcell) by intraperitoneal injection; (B) PDT-DC group: PDT-DC vaccines (approximately 1 × 10^6^ PDT-DCs in 0.1 ml PBS) were injected subcutaneously around peritumoral regions and 100 µg/mouse of Rat IgG2b isotype by intraperitoneal injection; (C) anti-PD-L1 mAb group: 0.1 ml PBS were injected subcutaneously around peritumoral regions and 100 µg/mouse of anti-PD-L1 mAb (10 F.9G2, BioXcell, West Lebanon, NH) by intraperitoneal injection; (D) combination group: PDT-DC vaccines were injected subcutaneously around peritumoral regions and 100 µg/mouse of anti-PD-L1 mAb by intraperitoneal injection. Tumor volumes were monitored using a caliper every three days. Mice were sacrificed when the tumors became necrotic or exceeded 2000 mm^3^, and serum was obtained for IL-12 and IL-6 measured by ELISA kit (Meimian industrial Co., Ltd, Jiangsu, China). All experiments were performed under an institutionally approved animal protocol.

### Immunohistochemistry

Tumor tissues, hearts, lungs, livers, spleens, and kidneys were dissected from mice. Specimens were fixed with 4% paraformaldehyde solution for 24 h at room temperature, then prepared by dehydration, paraffin dipping and embedding, cut into sections, and finally staining with Hematoxylin and Eosin (H&E). Tissue sections were incubated with 2% BSA for 30 min at room temperature, and immunohistochemical staining was performed on them with the primary antibodies and then secondary antibodies. The specimens were observed under BX53 fluorescence microscope (Olympus, Japan).

### Statistical analysis

All experiments were repeated at least three times. An unpaired t-test was applied for single comparisons between two groups for both in vitro and in vivo experiments. All data are expressed as the mean ± SD. One-way ANOVA was used among multiple comparison groups. Statistical analysis was performed using SigmaStat software (SPSS v20; IBM, Armonk, NY, USA). *P*< 0.05 was considered statistically significant.

## Results

### Cellular uptake of m-THPC in SCC7 cells

**Fig. 1:**
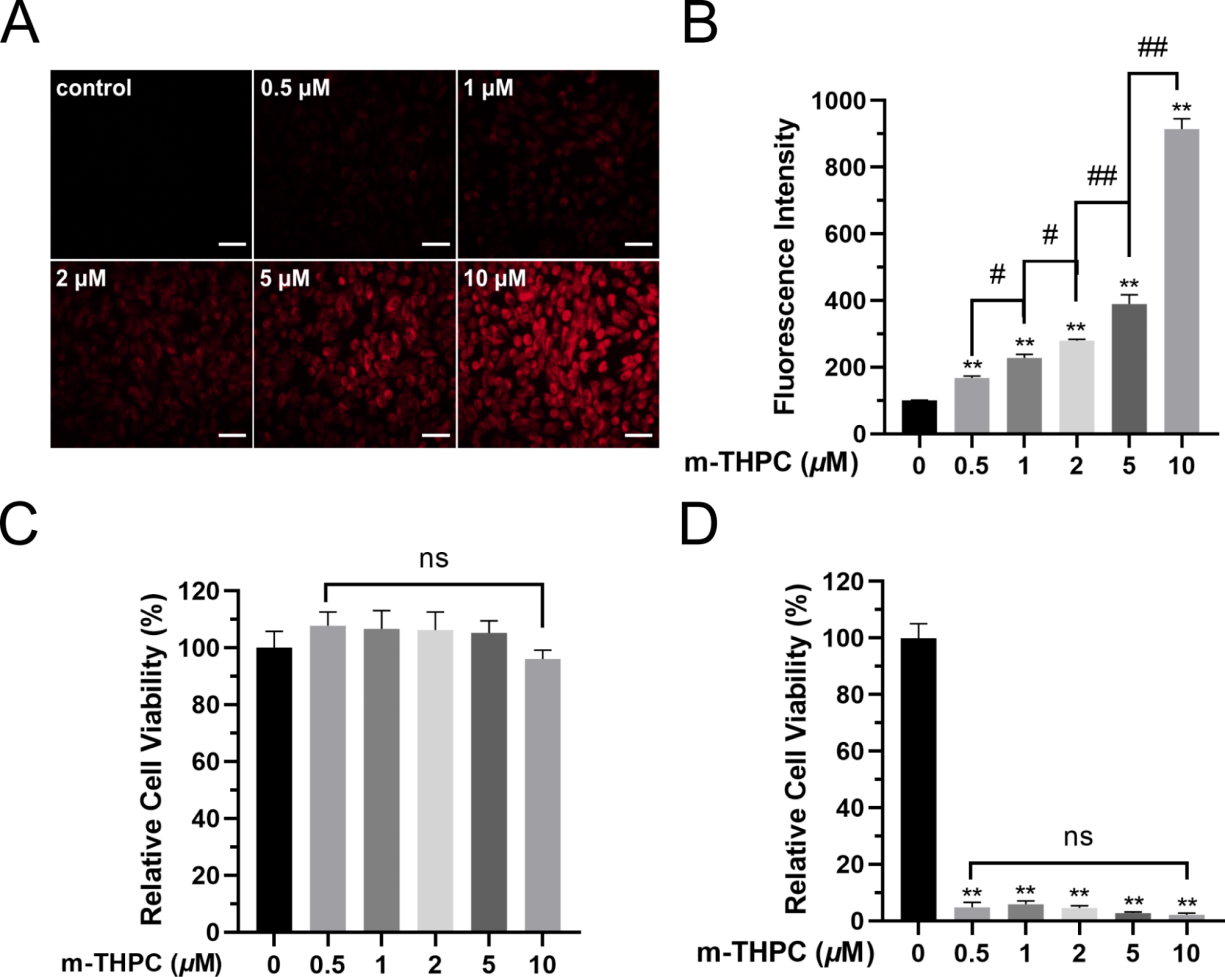
**Accumulation of m-THPC in SCC7 cells after incubation for 24 h.** (A) Detection by an inverted fluorescence microscope, the increased red fluorescence was observed with the increased amount of m-THPC (0.5 to 10 *µ*M) in SCC7 cells (Scale Bars = 20 *µ*m). (B) FI was measured at 650 nm using a microplate reader, high FI was observed when increasing the dose of m-THPC. (C) SCC7 cells were only treated with m-THPC (0.5 to 10 *µ*M) for 24 h, cell viability was determined by CCK-8 assay. (D) After incubation with m-THPC (0.5 to 10 *µ*M), SCC7 cells were treated with a fixed light dose (1 J/cm²), and cell viability was tested by CCK-8 assay. The results were obtained from three independent experiments and expressed as the mean ± SD. ^*^P < 0.05, ^**^P < 0.01 vs. control; ^#^*P* < 0.05, ^##^*P* < 0.01 among the groups. FI, fluorescence intensity.

SCC7 cells were incubated with different concentrations of m-THPC (0.5 to 10 *µ*M) in a serum-free medium in the dark for 24 h, and analyzed for fluorescence emission first using an inverted fluorescence microscope and then quantified using a microplate reader. The fluorescence intensity increased with m-THPC concentrations (Fig. 1 A and 1B). Furthermore, CCK-8 assay was used to evaluate cytotoxicity of m-THPC in SCC7 cells. m-THPC had no effect on cell viability (Fig. 1C); however, a decrease of cell viability was observed when cells were treated at a fixed light dose, which suggested that cell death was induced in SCC7 cells by treatment with PDT (Fig. 1D). Interestingly, m-THPC concentration was not a factor influencing cell viability when treating SCC7 cells with a fixed light dose, but significant suppressions of SCC7 cells across the range of m-THPC concentrations tested were observed (Fig. 1D). Taken together, 0.5 *µ*M m-THPC was selected as the experimental condition, because it has a similar effect on cell viability to different concentrations under the same light dose.

### PDT induces ICD in SCC7 cells

**Fig. 2:**
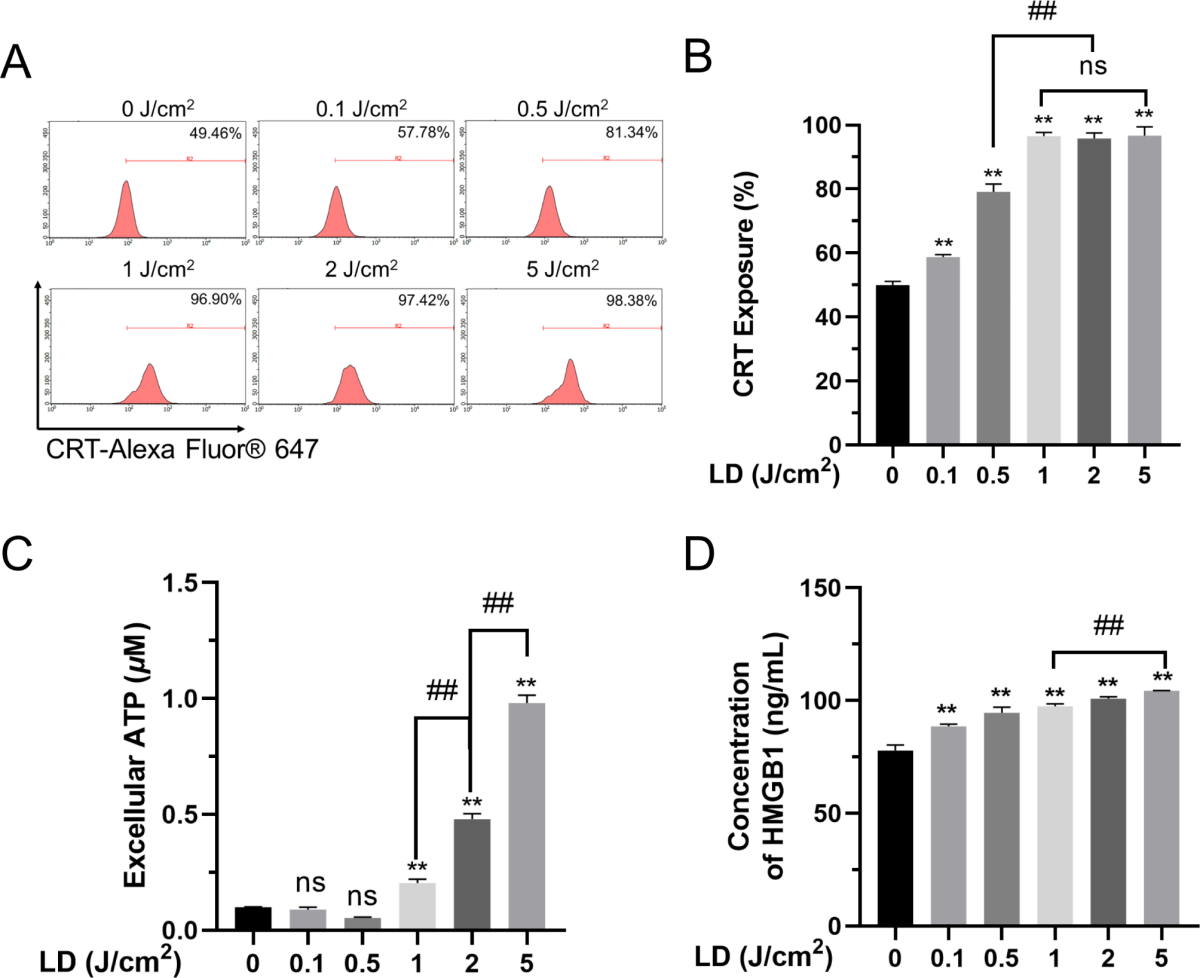
**ICD in SCC7 cells induced by m-THPC-PDT is associated with DAMPs.** (A) and (B) Quantification of flow cytometry analysis of CRT exposure at the cell surface. SCC7 cells were incubated 1 h after the treatment with PDT at different LD (0.1 to 5 J/cm²) (n = 3). (C) SCC7 cells were incubated for 4 h after PDT treatment and ATP was measured in the supernatants (n = 3). (D) SCC7 cells were incubated for 24 h after PDT treatment and HMGB1 was measured in the supernatants by ELISA (n = 3). The results were obtained from three independent experiments and expressed as the mean ± SD. ^**^P < 0.01 vs. control, ^##^*P* < 0.01 among the groups. LD: light dose.

It is known that PDT induces diverse types of cell death[32] depending on the concentration of photosensitizer and the light intensity[33]. To explore the optimal light dose of m-THPC-PDT induced ICD, we analyzed the presence of extracellular DAMPs, which induce innate immune response[34–36], a common feature of ICD. In addition, we measured the expression of CRT on the cell surface, and release of ATP and HMGB1 into the extracellular space[25, 37]. Immunofluorescent staining with an anti-CRT antibody showed that CRT exposure occurred as early as 1 h after PDT (Fig. 2A and 2B). Notably, CRT levels on the cell surface increased significantly at the low light dose groups (0.1 and 0.5 J/cm^2^) (Fig. 2A and 2B). High light doses caused the highest expression of CRT, but no further rise in cell surface CRT levels occurred at 1 J/cm^2^, 2 J/cm^2^, and 5 J/cm^2^ of light. We also analyzed the release of ATP (Fig. 2C) and HMGB1 (Fig. 2D) induced by PDT. In contrast to the CRT expression, no significant ATP release was noted at the low light doses (0.1 and 0.5 J/cm^2^). A significant rise in level of ATP release was observed at 1 J/cm^2^, which continued to increase in light dose dependent manner. For HMGB1, a significant increase in the extracellular medium occurred at the lowest dose of 0.1 J/cm^2^ which continued to increase up to 5 J/cm^2^. Basing on these observations, we considered that 5 J/cm^2^ of light dose and 0.5 *µ*M of m-THPC as the best doses for inducing optimal level of ICD in the SCC7 cells.

### PDT-activated ICD of SCC7 induces phenotypic and functional maturation of imDCs

To determine if ICD alters the phenotypic and functional maturation of mouse bone marrow imDCs, we analyzed surface expression of MHC-II in imDCs and patterns of IL-12 and IL-6 production, after coculturing with PDT-treated SCC7 cells. We compared imDCs co-cultured with SCC7 cells treated with m-THPC-PDT to those that were exposed to the freeze-thawed (F/T) cell lysates of SCC7 cells (F/T-SCC7).

**Fig. 3:**
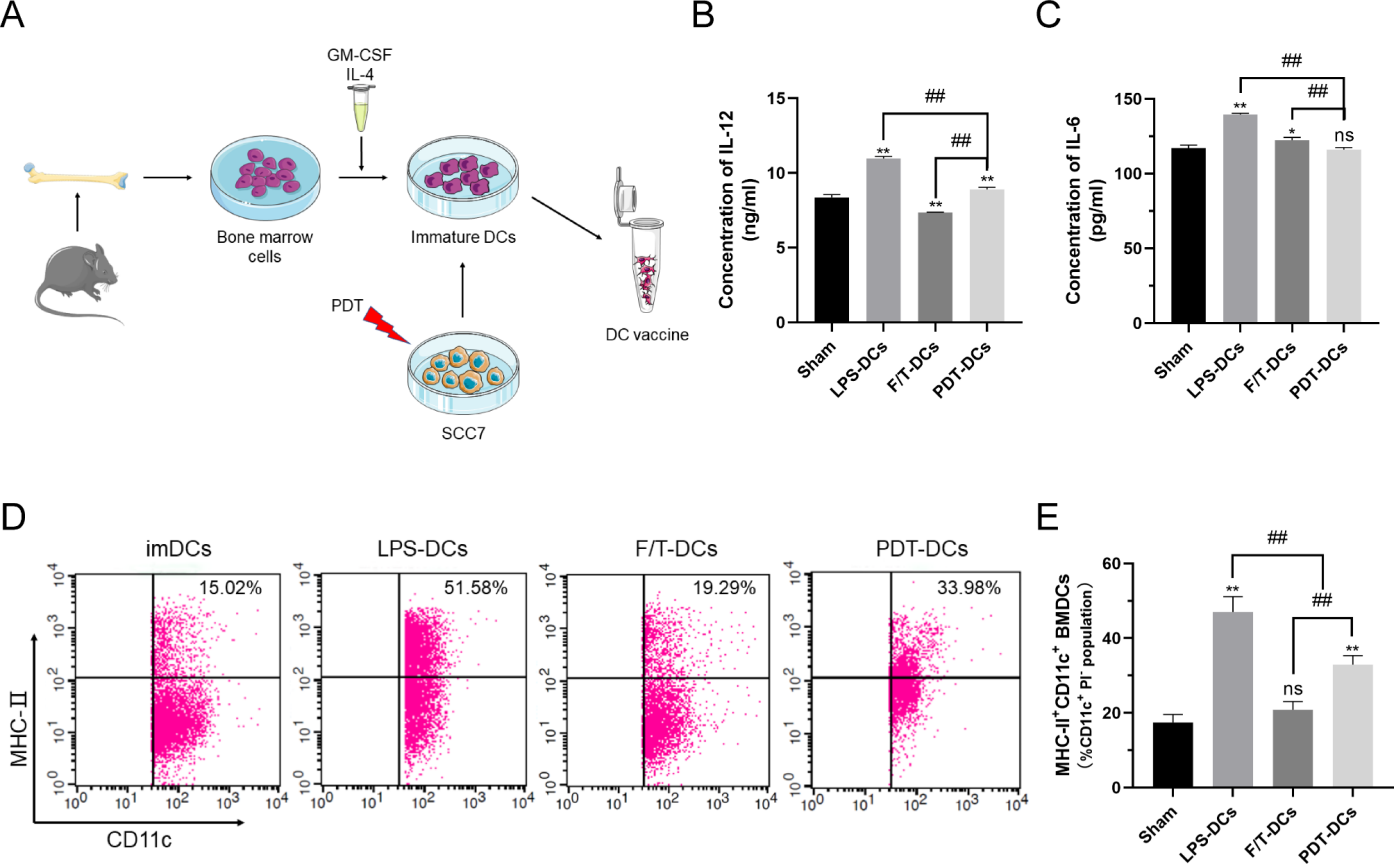
**Analysis of imDCs maturation in vitro.** (A) Generation of PDT-stimulated DCs. (B) Quantification of IL-12 production in the supernatants of imDCs were measured by ELISA (n = 3). The data from various treatment groups were plotted. (C) This panel is similar to panel B that the IL-6 production was measured by ELISA (n = 3). (D) and (E) Phenotypic maturation into DCs. imDCs were exposed for 24 h to SCC7 cells that were either pre-treated with m-THPC-PDT treatment or three cycles of F/T SCC7 cells, followed by the detection of MHC-II molecules on the surface of DCs. Cells double positive for CD11c and MHC-II were analyzed. The results were obtained from three independent experiments and expressed as the mean ± SD. ^*^P < 0.05,  ^**^P < 0.01 vs. control, ^##^*P* < 0.01 among the groups. DCs: dendritic cells; imDCs: immature DCs; F/T: freeze and thaw.

LPS- and sham-treated imDCS were used as positive and negative controls for these studies. As expected, imDCs stimulated with m-THPC-PDT displayed a markedly higher level of IL-12 than that stimulated by F/T SCC7 cells (Fig. 3B). In contrast, mTHPC-PDT treated group had a distinctly lower level of IL-6 (Fig. 3C). The LPS-treated cells had significantly higher levels of both IL-6 and IL-12. Similarly, we also found that the LPS-treated imDCs had the highest expression of MHC-II expression on their surface. The m-THPC-PDT treated SCC7 cells caused phenotypic maturation of imDCs, as indicated by the increase of MHC-II molecules on the cell surface, which was significantly higher than those treated with F/T-SCC7 (Fig. 3D and 3E). Collectively, these findings demonstrate that m-THPC-PDT potently induced the phenotypic and functional maturation of imDCs.

### PDT-DC vaccine enhances tumor regression induced by anti-PD-L1 mAb in vivo

**Fig. 4:**
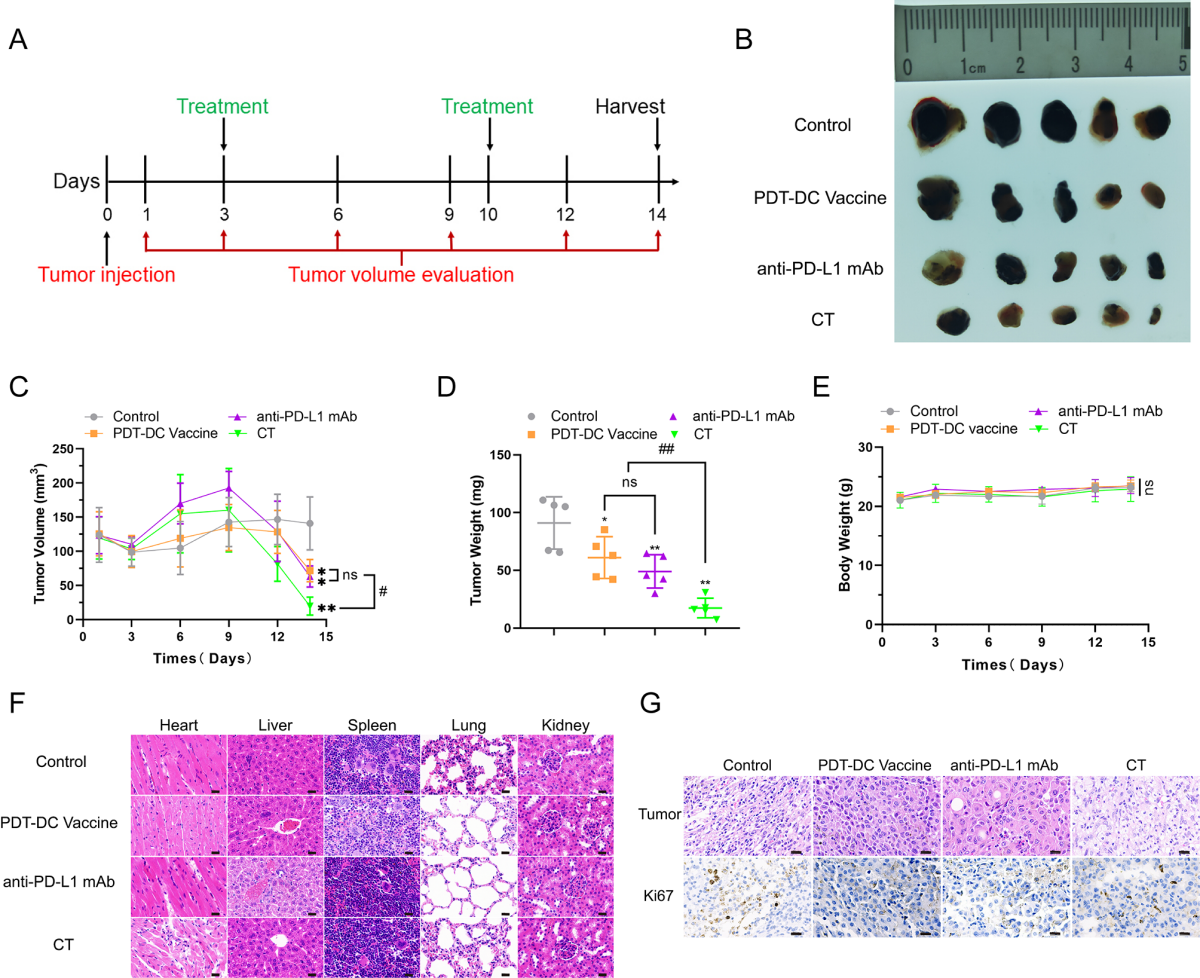
**PDT-DC vaccine enhances tumor regression induced by anti-PD-L1 mAb in vivo.** (A) Schematic representation of treatment protocol for HNSCC model. (B) Images of the resected tumors. (C) Tumor growths in mice were measured every three days for each group. (D) Tumor weights after 14days of various treatments. (E) The body weights of mice were measured every three days for each group. (F) H&E staining of the heart, liver, spleen, lung, and kidney on the 14th day after treating with the indicated agents (scale bars = 25 *µm*). (G) IHC analyses of tumor tissues for the expression of Ki67 (scale bars = 25 *µm*). ^*^P <  0.05, ^*^^*^P <  0.01 vs. control;  ^#^*P* < 0.05, ^##^*P* < 0.01 among the groups. CT: combination treatment.

Treatment with anti-PD-L1 antibody alone imparts significant therapeutic benefits. To explore if PDT-DC vaccine facilitated augments anti-tumor effects of anti-PD-L1 mAb in vivo, we used immunocompetent C3H/HeN mice bearing a subcutaneous SCC7 transplant (Fig. 4A). An isotypic control IgG was used as a negative control, which did not exert any anti-tumor effects. C3H/HeN mice immunized with PDT-DCs or anti-PD-L1 mAb alone significantly reduced tumor growth compared to the IgG control. However, the mice that received the combination treatment had the lowest tumor burden (Fig. 4B). The combination treatment strongly reduced tumor volume and tumor weight compared to either drug alone (Fig. 4C and 4D). The body weights of mice treated with the combination was not significantly different from the controls (Fig. 4E). Histopathological analyses did not reveal any significant damage to the heart, lung, spleen, kidney and liver following the various treatments used in this study (Fig. 4F). IHC analysis was used to confirm the changes in expression of growth-related markers. As shown in Fig. 4G, cell proliferation index Ki-67 shown no differences among these treatments.

### PDT-DC vaccine promoting the anti-tumor effects of anti-PD-L1 mAb by activating immune system

**Fig. 5:**
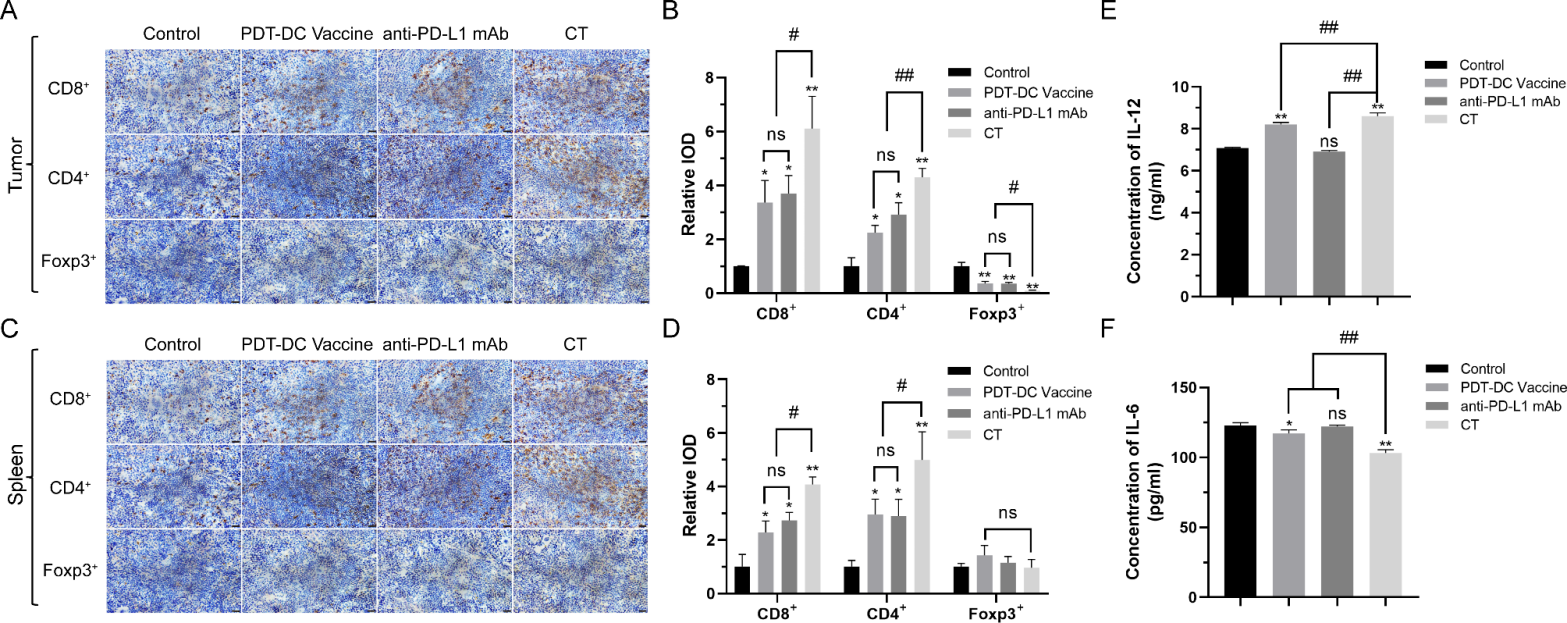
**PDT-DC vaccine promoting the anti-tumor effects of anti-PD-L1 mAb by activating immune system.** (A) and (B) IHC analyses of tumor tissues for the presence of CD8^+^, CD4^+^and Foxp3^+^ cells (scale bars = 20 *µm*). (C) and (D) IHC analyses of spleen sections for the CD8^+^, CD4^+^and Foxp3^+^ cells after treating the mice with the indicated agents (scale bars = 20 *µm*). (E) and (F) Serum levels of IL-12 and IL-6 in mice treated with the indicated agents. Cytokines were measured using ELISA (n = 3). The results of serum levels of IL-12 and IL-6 were obtained from three independent experiments and expressed as the mean ± SD. ^*^P < 0.05, ^**^P < 0.01 vs. control, ^#^*P* < 0.05,  ^##^*P* < 0.01 among the groups. CT: combination treatment.

The focus of tumor immunotherapy is to restore the killing activity of CTLs. To investigate the reactivation of immune response by the combination treatment, tumors and spleens from treatment groups were tested for the presence of increased numbers of anti-tumor T-cells using specific antibodies. CD8^+^ and CD4^+^ T cell numbers in tumors increased dramatically in combination treatment group while shown a decrease of Treg cells (Foxp3^+^) when compared with other groups in the tumor (Fig. 5A and 5B). A similar analysis of spleens showed an increase in CD8^+^ and CD4^+^ T cells in tumors, with no significant differences in Treg cells for every group (Fig. 5C and 5D). Additionally, secretion of inflammatory factors is crucial for immune system activation. Sera from the mice were also tested for changes in the levels of IL-12 and IL-6. Combination therapy significantly increased serum IL-12 levels. In contrast, IL-6 levels were lower in mice that received the combination therapy. These observations are consistent with those of in vitro studies (Fig. 5E and 5F).

## Discussion

Growing evidence suggests that HNSCCs are insensitive to immunotherapies due to high load of mutated neoantigens[31], which promotes to immune evasion directly and indirectly through some uncertain mechanisms[38]. For example, HNSCCs can secret some immunosuppressive cytokines to impede antigen recognition and tumor killing of T cells[39–41], and also deplete nutrients in tumor microenvironment (TME) inhibiting T cell proliferation and activation[40]. The upregulation of PD-L1 on the surface of HNSCC plays a crucial role in inducing apoptosis of tumor infiltrating lymphocytes and hindering differentiation of CTLs, which appears to be a major mechanism for immune escape of HNSCCs[42]. Notably, exosomes derived from HNSCCs are also carried with immunosuppressive substances such as PD-1 and cytotoxic T lymphocyte antigen 4 to impair anti-tumor immunity[43, 44]. Classical HLA class I antigen loss/downregulation of HNSCCs is associated with tumor immune escape leading to abnormalities of antigen-presentation to CTLs[45]. In addition to those immunosuppressive mechanisms, some co-stimulatory molecules and pathways of immune evasion were identified in HNSCCs [46–48]. Immunosuppressive cell populations are key component in immune system, while HNSCCs recruit immunosuppressive cell such as Tregs into TME to inhibit CTLs[49, 50].

The success of immune checkpoint inhibitors especially anti-PD-L1 mAb has been considered as a promising way for controlling many kinds of tumors and highlights the specific role of T cells in immunotherapy. Unfortunately, many clinical trials confirmed that high level of PD-L1 expression on the surface of cancer cells does not mean a high response rate to anti-PD-L1 mAb[51, 52]. Moreover, longterm use of anti-PD-L1 mAb also has an undesirable side effect such as an autoimmune-like damage of normal tissues because of a global non-specific activation of other CTLs. A therapy that more effectively activates CTLs to selectively kill tumor cells would help alleviate this problem.

PDT may be a potential approach for some solid tumors where an interaction between a photosensitizer and a specific wavelength of light leads to immunogenic tumor death[53]. This process contributes to a generation of intracellular ROS (in the tumor) which cause cytotoxicity. Accumulating evidence supports the notion that cancer cells undergo ICD, which potently stimulates the APCs unlike other types of cell deaths, and is associated with the release of DAMPs into the TME [25]. Notably, ICD uniquely stimulates DC maturation because it provides specific tumor associated antigens.

We considered that the PDT sensitized DC vaccine may present specific HNSCC associated antigens to CTLs which were activated by anti-PD-L1 mAb. Likewise, this kind of DC vaccine is able to overcome limitations of application of anti-PD-L1 mAb.

Indeed, the combination of m-THPC and a light dose were able cause the release of DAMPs and potently stimulated imDCs to differentiate in our experiment (Figs. 2 and 3). ICD is characterized by CRT, ATP and HMGB1 emission. The exposure of CRT on the cell membrane is the most common trait of PDT-induced tumor cells ICD, which is caused by endoplasmic reticulum stress during PDT[54]. Although the underlying mechanisms of CRT exposure are different depending on the type of photosensitizer used [23, 55], the phosphorylation of eukaryotic initiation factor 2α is universally considered to be necessary for CRT exposure[56]. The release of ATP under PDT has its specific mechanism like CRT[57]. In dying tumor cells, ATP was secreted via classical endoplasmic reticulum/Golgi secretory pathway[55]. As a DAMP, HMGB1 is passively secreted into external environment from PDT-induced ICD tumor cells[58, 59], while the mechanism of this process was not fully understood. It is notable that PDT-induced ICD was modulated by photosensitizer and light dose. These two factors affect the strength of PDT-induced ICD and determine how quickly when tumor cells reaching the late stages of cell death, and also decisive factors for immunogenicity of ICD tumor cells[60]. This may be a possible explanation for the trend of CRT, ATP, and HGMB changes under different light dose in our experiment.

As a subgroup of antigen-presenting cells, DCs are responsible for priming anti-tumor immunity through antigens presentation and stimulation of primary T cells. High expression of MHC-II molecules, a commonly used marker of DC cell morphological maturation, is responsible for presenting tumor specific antigens to T cells. Meanwhile, DCs can release some inflammatory cytokines as the third signal to facilitating T cell maturation. IL-12 plays a predominant role in T-cell polarization[61]. IL-6 is traditionally regarded as a pro-inflammatory cytokine, while some recent studies have found that IL-6 also exhibits anti-inflammatory properties in some cases[62]. High level of IL-6 secreted by DCs suppresses the expression of MHC-II molecules and co-stimulatory molecules on immature DCs[63]. Additionally, IL-6 secretion by DCs also induce immature DCs into a semimature state and finally prevent T cells activation[64–67].

Currently, a number of stimulants (such as chemokines[12], tumor antigen fusion protein[13], and tumor antigen peptide[14]) have been proved good stimulations for DC vaccines preparation. F/T-tumor cells were the most common used method to obtain those stimulants. We compared stimulating effects of F/T-tumor cells and PDT-induced ICD tumor cells on DCs maturation intentionally. As shown in Fig. 3, in terms of stimulating morphological and functional maturation of DCs, PDT-induced ICD tumor cells were far superior to that of F/T-tumor cells. DCs stimulated by PDT can recognize antigens of squamous cell carcinoma and present these antigens to CTLs. In vivo, the combination treatment not only reduced the tumor proliferation stronger than the controls (Fig. 4), but also increased a high level of CD8^+^ and CD4^+^ T cells infiltration. Simultaneously, we also noted a decrease of immunosuppressive Tregs in the tumor. In addition, the combination treatment also affected the anti-tumor immune response in other organs, as observed by a rise of the CD8^+^ and CD4^+^ T cells in the spleen, increasing the secretion of pro-inflammatory cytokine IL-12, and decreasing the secretion of pro-tumor inflammatory factor IL-6 (Fig. 5). It is also noteworthy that the combination treatment has no damage of five normal tissues (heart, liver, spleen, lung, and kidney) in the tumor-bearing mice according to histochemical analysis (Fig. 4).

In summary, we demonstrated that PDT sensitized DCs can robustly promote the anti-tumor effects of anti-PD-L1 mAb in a murine model. Moreover, the current study reveals the application of a DC vaccine as an adjunct since it was commonly used as the single method in immunotherapy.

## Data Availability

All data that support the findings of this study are available from the corresponding authors upon reasonable request.

## List of abbreviations

HNSCC: head and neck squamous cell carcinoma
CTL: cytotoxic T-lymphocytes
DC: dendritic cell APC antigen presenting cells
PDT: photodynamic therapy
ROS: reactive oxygen species
ICD: immunogenic cell death
DAMPs: damage-associated molecular patterns
mAb: monoclonal antibody
CRT: calreticulin
HMGB1: high mobility group protein B1
F/T: freeze-thawed
LPS: *E. coli* lipopolysaccharide
Interleukin-12: IL-12
Interleukin-6: IL-6
TME: tumor microenvironment
